# Modern hysteroscopic management of congenital uterine anomalies: current techniques, emerging technologies, and future directions

**DOI:** 10.3389/fsurg.2026.1931279

**Published:** 2026-10-01

**Authors:** Mariana da Cunha Vieira, Giovanna C. C. Martin, Katherine A. R. Miller, Juliana A. Oliveira, Ana Clara P. Servidoni, Mauricio S. Abrao

**Affiliations:** 1 Gynecology Division, Beneficência Portuguesa de São Paulo, São Paulo, Brazil; 2 Department of Medicine, Hospital Santa Marcelina, São Paulo, Brazil; 3 Department of Medicine, University Santo Amaro, São Paulo, Brazil; 4 Department of Surgery, Federal University of Minas Gerais, Belo Horizonte, Brazil; 5 Gynecology Division, Obstetrics and Gynecology Department, Faculdade de Medicina da Universidade de São Paulo, São Paulo, Brazil

**Keywords:** dysmorphic uterus, hysteroscopy, metroplasty, minimally invasive gynecology, Müllerian anomalies, reproductive outcomes, Robert's uterus, septate uterus

## Abstract

**Background:**

Congenital uterine anomalies are clinically relevant in reproductive medicine, particularly among women with infertility, recurrent pregnancy loss, adverse obstetric outcomes, or obstructive symptoms. Hysteroscopy has transformed the management of correctable intracavitary anomalies by enabling conservative, image-guided reconstruction of the uterine cavity while avoiding the morbidity of historical abdominal metroplasty.

**Scope:**

This narrative review critically examines contemporary hysteroscopic management of septate uterus, dysmorphic uterus, bicorporeal septate uterus and rare obstructive malformations, such as Robert's uterus. We discuss the implications of the ESHRE/ESGE, CUME, and ASRM classification systems; the role of three-dimensional transvaginal ultrasound and magnetic resonance imaging in preoperative mapping; and the technical principles that guide metroplasty, cavity remodeling, ultrasound-guided hysteroscopy, office-based metroplasty, and staged approaches in complex anatomy.

**Discussion:**

Bipolar saline-based platforms, miniaturized resectoscopes, bipolar needle electrodes, cold scissors, and selected tissue removal systems have expanded the therapeutic spectrum and improved safety. The evidence supporting surgery remains heterogeneous: the TRUST randomized trial challenged routine septum resection in unselected women, whereas data for T-shaped and other dysmorphic uteri remain largely observational. Emerging technologies, including laser platforms, artificial intelligence-assisted hysteroscopy, and robotic concepts, are promising, but currently lack prospective validation.

**Conclusion:**

Optimal management should therefore be individualized, anatomy-driven, and grounded in shared decision-making, with careful preservation of endometrium and myometrium. Standardized reporting, multicenter registries, and prospective comparative studies are needed to define which patients benefit most from hysteroscopic correction.

## 1 Introduction

Congenital uterine anomalies arise from abnormal development, fusion, or resorption of the Müllerian ducts and include a broad spectrum of uterine, cervical, and vaginal malformations. Their clinical relevance varies substantially: many anomalies are incidental, whereas others are associated with infertility, recurrent pregnancy loss, preterm birth, malpresentation, dysmenorrhea, hematometra, or obstructive symptoms. A recent meta-analysis estimated their prevalence at 5.5% in unselected populations, rising to 8% in infertile women, 13.3% in those with recurrent miscarriage, and 24.5% in women with both infertility and pregnancy loss (1, 2).

Hysteroscopy has fundamentally changed the surgical management of correctable intracavitary anomalies. Direct transcervical access allows the surgeon to restore a more physiological uterine cavity through septum incision, controlled lateral wall remodeling, or selected creation of communication in obstructive malformations. The reconstructive goal is not maximal tissue removal, but the minimum intervention required to achieve a functional triangular cavity, while preserving endometrium and surrounding myometrium (3, 4).

The field has evolved from monopolar resectoscopy performed with non-physiologic distension media to bipolar saline platforms, miniaturized instruments, office-based approaches, cold mechanical techniques, tissue removal systems, laser platforms, and image-guided strategies. In parallel, three-dimensional transvaginal ultrasound has become central to diagnosis and surgical planning by showing the internal cavity and external fundal contour simultaneously. Artificial intelligence and robotic concepts are also entering the discussion, although their role remains investigational (4–6).

This review summarizes current techniques, safety principles, ongoing controversies, evidence gaps, and future directions in the hysteroscopic management of congenital uterine anomalies. Particular emphasis is placed on individualized patient selection, anatomy-driven surgical planning, reproductive goals, and fertility-preserving uterine reconstruction, aiming to restore a functional uterine cavity while minimizing unnecessary disruption of healthy endometrial and myometrial tissue. At the same time, the therapeutic role of hysteroscopy remains limited or absent in the literature for anomalies without a correctable intracavitary component, such as unicornuate uterus, uterus didelphys, and Mayer–Rokitansky–Küster–Hauser (MRKH) syndrome.

## 2 Methods

A structured literature search was conducted in MEDLINE/PubMed, Embase, Scopus, and Web of Science from database inception to July, 2026. The search combined terms related to congenital uterine anomalies and hysteroscopic management, including *uterine anomalies*, *Müllerian anomalies*, *septate uterus*, *dysmorphic uterus*, *Robert's uterus*, *hysteroscopy*, and *hysteroscopic metroplasty*. Reference lists of relevant publications were also screened to identify additional studies.

As this was a narrative review, studies were selected based on relevance to classification, diagnosis, surgical techniques, perioperative safety, reproductive outcomes, and emerging technologies. The evidence was synthesized qualitatively. The full search strategy is available on supplementary material.

## 3 Classification of uterine anomalies

Several classification systems have been proposed, with the most widely adopted being those from the European Society of Human Reproduction and Embryology (ESHRE)/European Society for Gynaecological Endoscopy (ESGE), the Congenital Uterine Malformations by Experts group (CUME), and the American Society for Reproductive Medicine (ASRM). The value of a structured classification lies in systematizing the understanding of these anomalies and standardizing the identification of uterine, cervical, and vaginal malformations, thereby supporting accurate diagnosis, informed patient counseling, and more targeted treatment (3)**.**

### 3.1 ESHRE/ESGE classification

The 2013 ESHRE/ESGE classification system categorizes uterine (U0–U6), cervical (C0–C4), and vaginal (V0–V4) anomalies independently, allowing a comprehensive description of complex Müllerian malformations. Importantly, it introduced objective morphometric criteria based on uterine wall thickness: a septate uterus is defined by an internal fundal indentation exceeding 50% of the uterine wall thickness, whereas a bicorporeal uterus is characterized by an external fundal indentation exceeding 50% of the uterine wall thickness. The system also introduced the category of dysmorphic uterus (U1), encompassing T-shaped uterus, infantile uterus, and other dysmorphic variants characterized by a normal external fundal contour and an abnormal uterine cavity, excluding septate uterus (3).

This classification provides a systematic and anatomically coherent framework, particularly valuable for describing complex combinations of uterine, cervical, and vaginal anomalies. However, the use of uterine wall thickness as the reference denominator may increase the apparent prevalence of septate uterus, as this measurement can be influenced by factors such as adenomyosis, leiomyomas, and variations in overall myometrial thickness (1, 7). These limitations should therefore be taken into account when applying morphometric thresholds to clinical decision-making and surgical indication. **(**Supplementary Figure S1**).**

### 3.2 CUME classification

The Congenital Uterine Malformations by Experts (CUME) group proposed criteria intended to reduce disagreement in the diagnosis of septate uterus. CUME defines septate uterus primarily by an internal indentation depth greater than 10 mm. For T-shaped uterus, CUME proposed lateral indentation depth of at least 7 mm, lateral indentation angle of 130 degrees or less, and T-angle of 40 degrees or less (8, 9). These absolute measurements may improve reproducibility in borderline cases.

### 3.3 ASRM classification

The American Society for Reproductive Medicine (ASRM) Müllerian Anomalies Classification 2021 uses descriptive categories rather than the older numerical American Fertility Society (AFS) framework. The Septate uterus is defined by a septum extending more than 1 cm below the interostial line with an angle of less than 90 degrees, whereas the bicornuate uterus is defined by an external fundal indentation greater than 1 cm. The arcuate uterus is considered clinically insignificant and grouped with normal uterine morphology. Robert's uterus is included as a rare asymmetric obstructive form of septate uterus. Dysmorphic cavities such as T-shaped and Y-shaped uteri are not included as distinct categories, which remains controversial given the emerging, but heterogeneous literature on reproductive outcomes after metroplasty (10).

### 3.4 Clinical implications of classification choice

Classification choice directly influences diagnosis, counseling, and surgical decision-making, as the same uterine morphology may be categorized differently across classification systems and consequently alter whether hysteroscopic treatment is considered. However, morphometric criteria alone do not establish clinical relevance and should be interpreted alongside symptoms, reproductive and obstetric history, patient goals, and shared decision-making. This is particularly relevant following the TRUST randomized trial, which questioned routine septum resection in unselected women with a septate uterus (11–13) **(**Figure 1**)**. Importantly, the therapeutic role of hysteroscopy remains limited or absent in anomalies without a correctable intracavitary component, such as unicornuate uterus, uterus didelphys, and Mayer–Rokitansky–Küster–Hauser (MRKH) syndrome.

**Figure 1:**
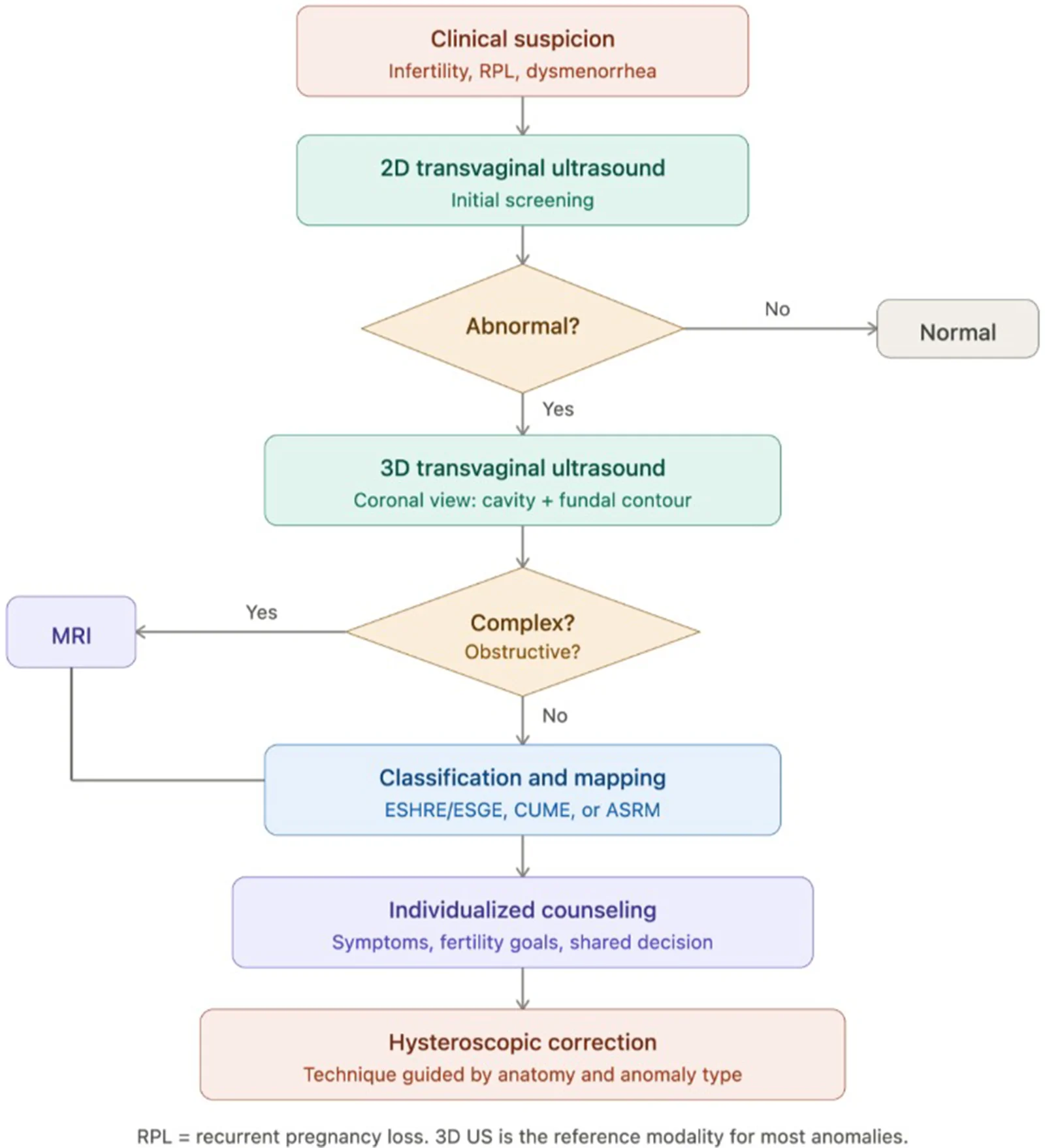
Diagnostic workup for symptomatic uterine anomalies.

## 4 Preoperative evaluation and anatomical mapping

### 4.1 Preoperative assessment as a determinant of surgical success

Accurate preoperative anatomical characterization is essential, once hysteroscopy provides an intracavitary perspective only. Hysteroscopy alone cannot reliably distinguish a septate uterus with preserved external fundal contour from a bicorporeal uterus with an external fundal cleft. Misclassification may expose a patient to an inappropriate procedure or deny surgery to a patient with a correctable anomaly. Preoperative assessment should confirm the diagnosis, define the three-dimensional geometry of the cavity and myometrium, evaluate associated cervical and vaginal anomalies and detect coexisting intrauterine pathology that may alter the surgical plan (14)**.**

### 4.2 Imaging modalities and three-dimensional mapping

Three-dimensional transvaginal ultrasound is the reference imaging modality for most congenital uterine anomalies because it provides a coronal view that simultaneously demonstrates the external fundal contour and the internal cavity. It enables measurement of indentation depth, indentation angle, lateral wall morphology, cavity width, and residual myometrial thickness, thereby supporting the ESHRE/ESGE, CUME, and ASRM diagnostic frameworks (1, 14).

Three-dimensional ultrasound can also be integrated into the operative workflow. In a prospective outpatient study of women with ESHRE/ESGE class U1 or U2 anomalies, preoperative three-dimensional ultrasound, see-and-treat hysteroscopy, and immediate postoperative ultrasound in a single visit improved cavity morphology and volume and reduced time to completed treatment (15). This supports a model in which imaging is not merely diagnostic, but also intraoperative and quality-control oriented.

Magnetic resonance imaging (MRI) should be reserved for complex or ambiguous anatomy, obstructive malformations, suspected associated pelvic pathology, or technically limited ultrasound. MRI offers a global pelvic assessment and excellent soft-tissue contrast, but it is more expensive, less dynamic, and less available than high-quality three-dimensional ultrasound (14).

### 4.3 Mapping coexisting intracavitary pathology

Preoperative evaluation should systematically identify submucosal myomas, polyps, adhesions, residual septal tissue after prior surgery, and endometrial abnormalities. Coexisting pathology may change the sequence, setting, instrument choice, and need for staging. A fundal submucosal myoma associated with a septum is particularly demanding, because both lesions compete for the same operative field and increase the cumulative vascular surface exposed to distension media **(**Figure 1**)**.

## 5 Surgical techniques for hysteroscopic correction

### 5.1 Correction of septate uterus

Hysteroscopic septoplasty is the conservative surgical approach for septate uterus when intervention is clinically justified. The objective is to incise the septum from its apex toward the fundus until the cavity assumes a symmetric triangular shape and both tubal ostia are visible in a panoramic view. The endpoint should be anatomical and functional, not simply based on the appearance of bleeding, because septa may contain fibromuscular tissue and variable vascularity (16).

The surgical endpoint is reached when the hysteroscope moves freely between both cornual recesses, the residual fundal indentation is minimal according to the chosen classification, and adequate myometrial thickness is preserved. In complete septa, broad-based septa, previously operated cavities, and uncertain anatomy, intraoperative ultrasound helps prevent over-incision and perforation (16, 17)**.**

#### 5.1.1 Conventional resectoscope (monopolar and bipolar)

Conventional resectoscopy remains widely used, especially for thick or complete septa. Bipolar systems using saline are now preferred, when available, because they avoid the electrolyte disturbances associated with monopolar surgery using non-physiologic media. The incision is generally performed in the midline from the caudal apex toward the fundus, with alternating cuts to maintain symmetry. For broad septa, controlled reduction on each side helps avoid deviation toward one uterine wall (18–21) **(**Figure 2**)**.

**Figure 2:**
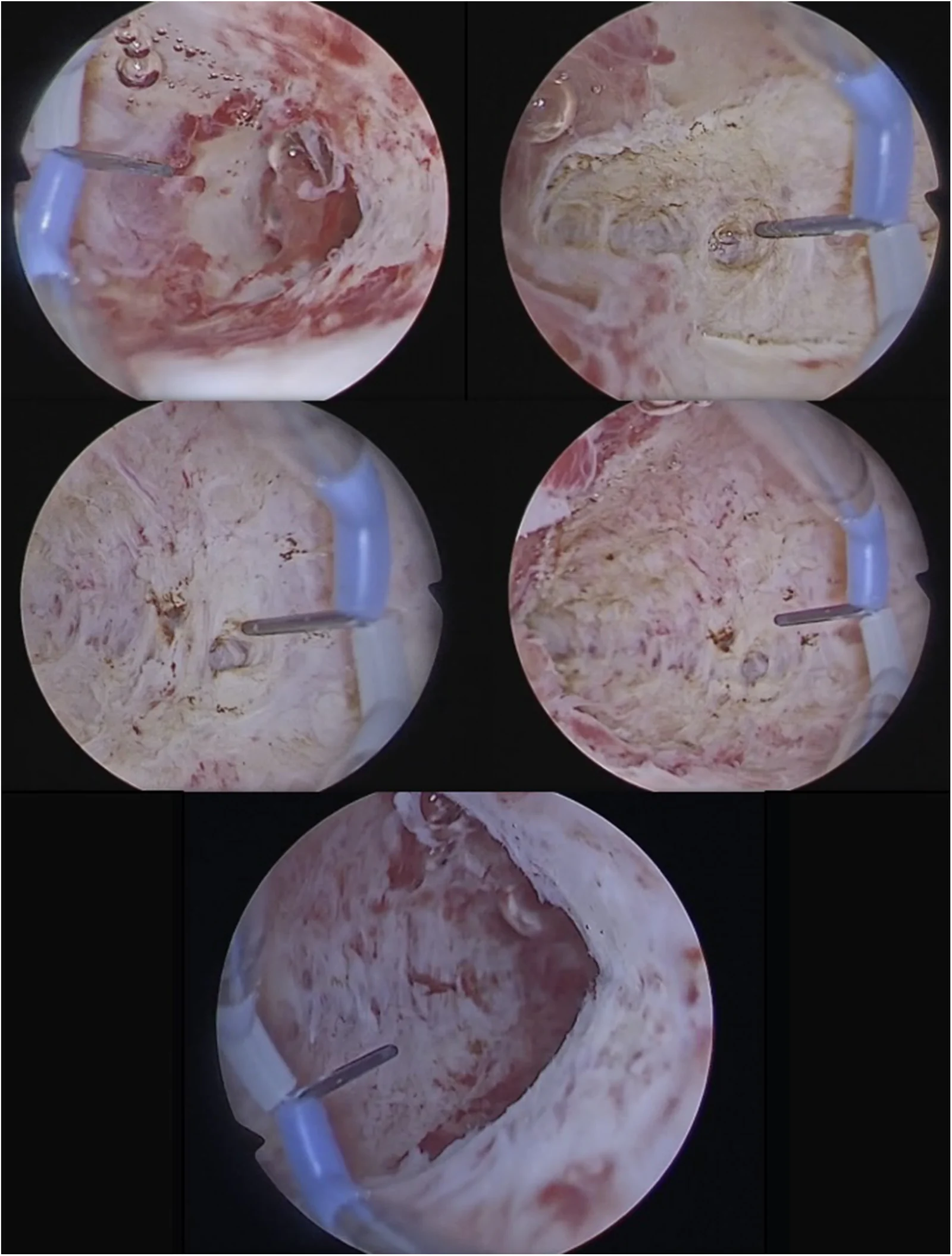
Conventional resectoscopy metroplasty. Hysteroscopic images demonstrating sequential steps in the surgical correction of a septate uterus. (**A-D**) Progressive resection of the uterine septum using a resectoscope with electrosurgical cutting. (**E**) Final view of the uterine cavity following complete septum resection and insertion of the anti-adhesion gel.

In complete uterocervical septa, the decision to divide or preserve the cervical component should be individualized. The traditional approach preserves the cervical canal and begins above the internal os, but complete division of the cervical septum has also been reported without clear evidence of increased cervical incompetence. The available evidence remains limited, and counseling should acknowledge uncertainty (22).

#### 5.1.2 Mini-Resectoscopy (15 Fr), miniaturized instruments, and office-based metroplasty

Miniaturized resectoscopes (15 Fr, 5 mm outer diameter), have enabled septum incision with less need for cervical dilatation and with the possibility of outpatient or office-based management in selected patients. Mini-resectoscopy can reproduce the principles of conventional resectoscopy while reducing access-related trauma and improving feasibility in narrow cavities. With miniaturized instruments, resection begins at the apex, usually with a bipolar electrode operated in pulsed mode, working alternately from one side of the septum to the other; after approximately three-quarters of the septum has been removed, the bipolar electrode can be replaced by miniature scissors to achieve a neat finish and to trim the residual fundal tissue (22, 23) **(**Figure 3**)**.

**Figure 3:**
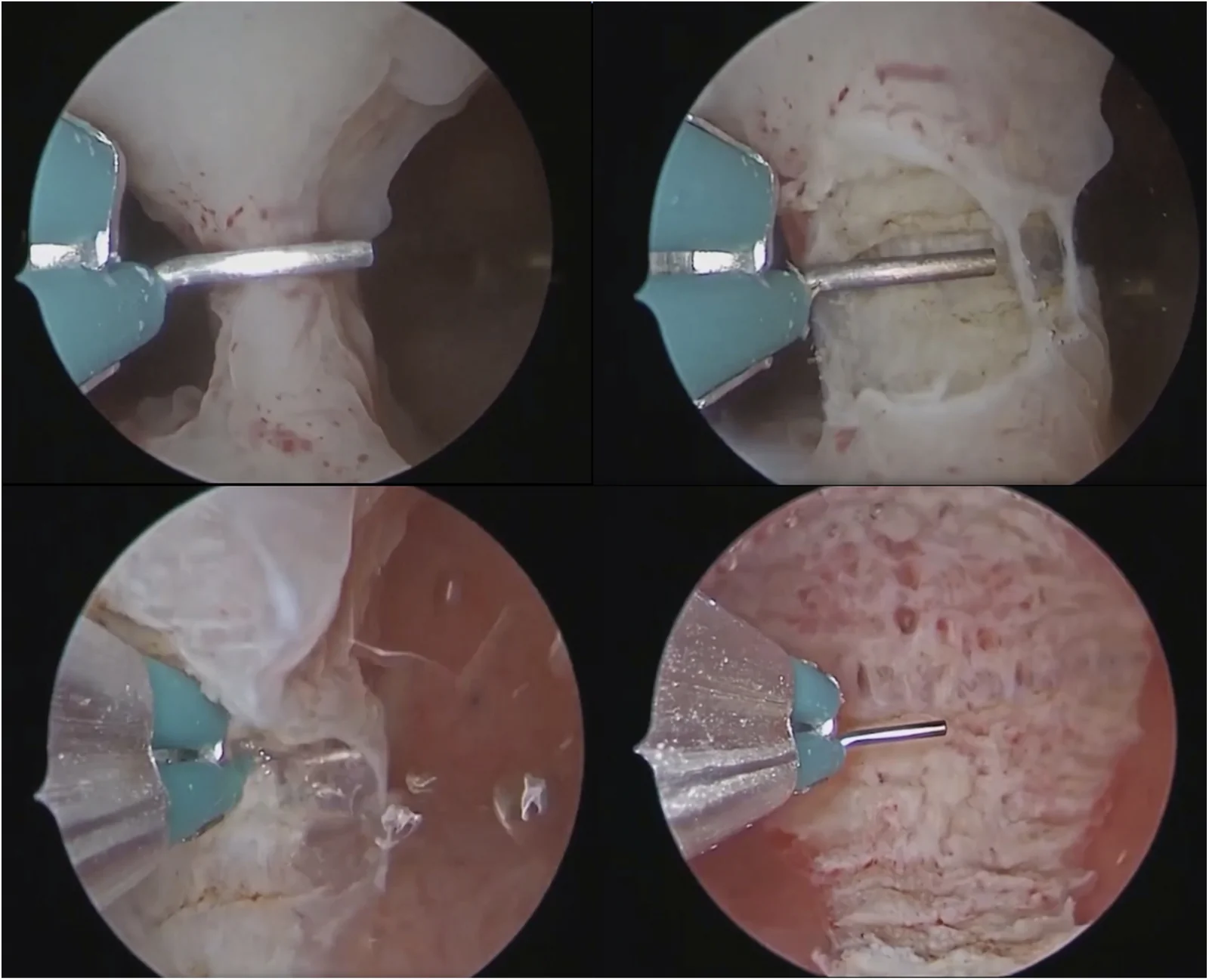
Mini-resectoscope metroplasty. Hysteroscopic images demonstrating sequential steps in the surgical correction of a septate uterus using a mini- resectoscope and Collins electrode. (**A-C**) Progressive resection of the uterine septum from its apex toward the fundus. (**D**) Final view of the uterine cavity following complete septum resection and insertion of the anti-adhesion gel.

A modified metroplasty technique using the 15 Fr bipolar office resectoscope, uses a Collins electrode to make the incision of the septum and then resects the fibromuscular tissue from the anterior, posterior, and fundal walls until the central muscular component is removed (16, 23). This approach reflects the rationale as the histologic study by Fascilla et al. (16), in which the septum was excised as whole triangular strips from the fundus to the apex to characterize its full three-dimensional structure (16) **(**Figure 4**)**.

**Figure 4:**
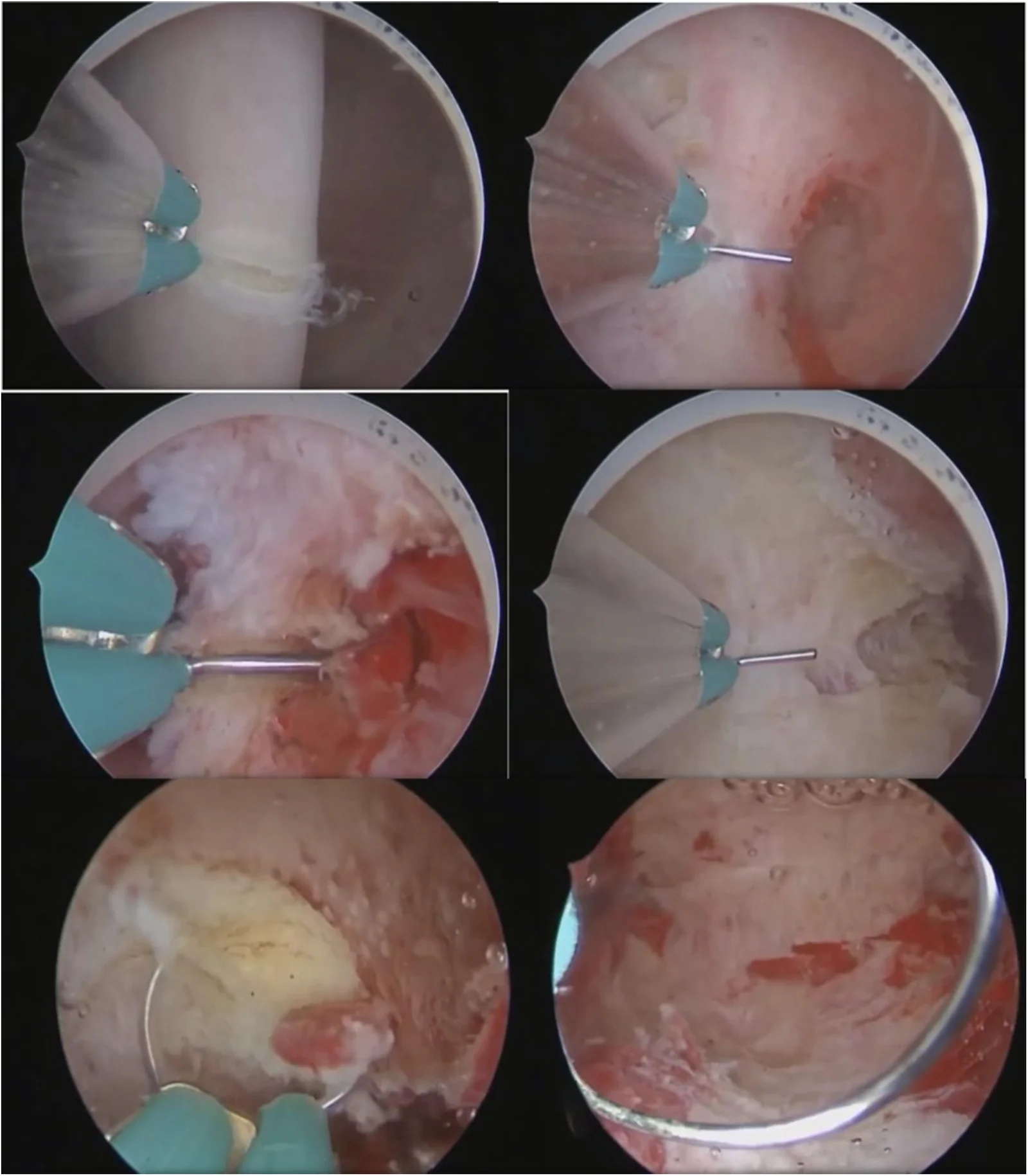
Modified metroplasty technique using a 15 Fr bipolar office resectoscope. Hysteroscopic views demonstrating key steps: **(A)** Collins electrode incising the vaginal septum; **(B,C)** Collins electrode incising the cervical septum; **(D)** Progressive resection of the fibromuscular septal tissue from the anterior, posterior, and fundal walls; **(E,F)** Completed resection showing the muscular component removed and a unified cavity.

#### 5.1.3 Bipolar needle electrode and office hysteroscopy

The bipolar needle or twizzle electrode inserted through a continuous-flow diagnostic hysteroscope allows precise incision with limited tissue effect. When combined with the vaginoscopic no-touch technique, it can be performed in an office setting or under minimal anesthesia in selected patients. In a prospective randomized study, small-diameter hysteroscopy with a bipolar system was associated with shorter operative time, less fluid absorption, and fewer complications than monopolar resectoscopy, with comparable reproductive outcomes (6). A intrauterine palpator instrument may be used in the final stages to measure the actual depth of metroplasty and improve accuracy (15, 23) **(**Figure 5**)**.

**Figure 5:**
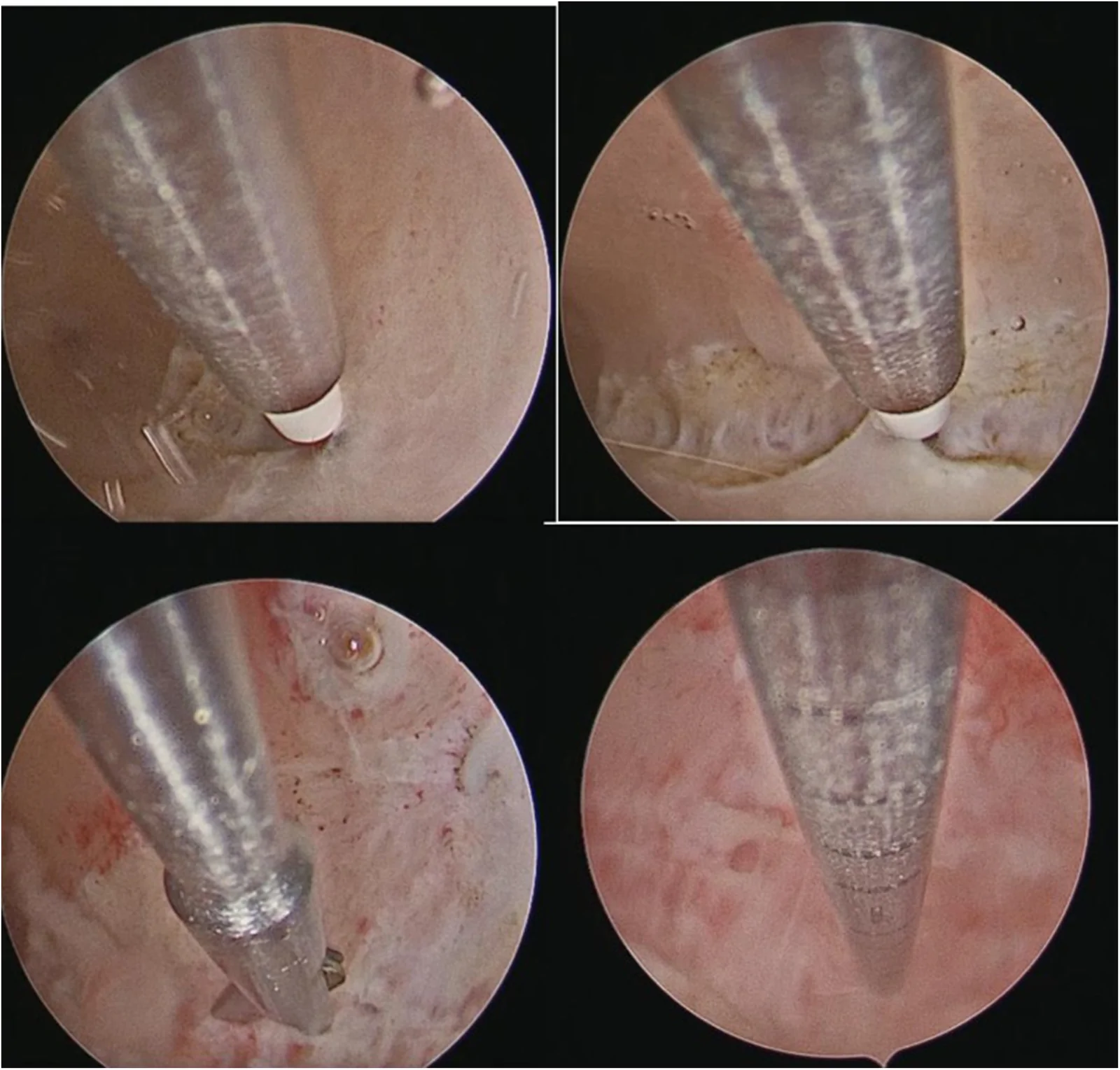
Office hysteroscopic metroplasty. (A, B) Bipolar needle makes precise septal incision through small-diameter hysteroscope, enabling an outpatient no-touch procedure; (C) The final portion of the septum is incised with miniaturized scissors. (D) Intrauterine 5-Fr palpator confirms complete resection.

#### 5.1.4 Cold scissors metroplasty

Cold scissors avoid thermal injury and are particularly attractive for thin or partial septa, young patients, and fertility-preserving reconstruction when minimizing endometrial damage is a priority. Their principal limitation is reduced hemostatic capacity in thick, vascular, or fibromuscular septa. Instrument choice should therefore be guided by septal thickness, vascularity, cavity size, surgical setting, and surgeon experience (17, 20) **(**Figure 6**)**.

**Figure 6:**
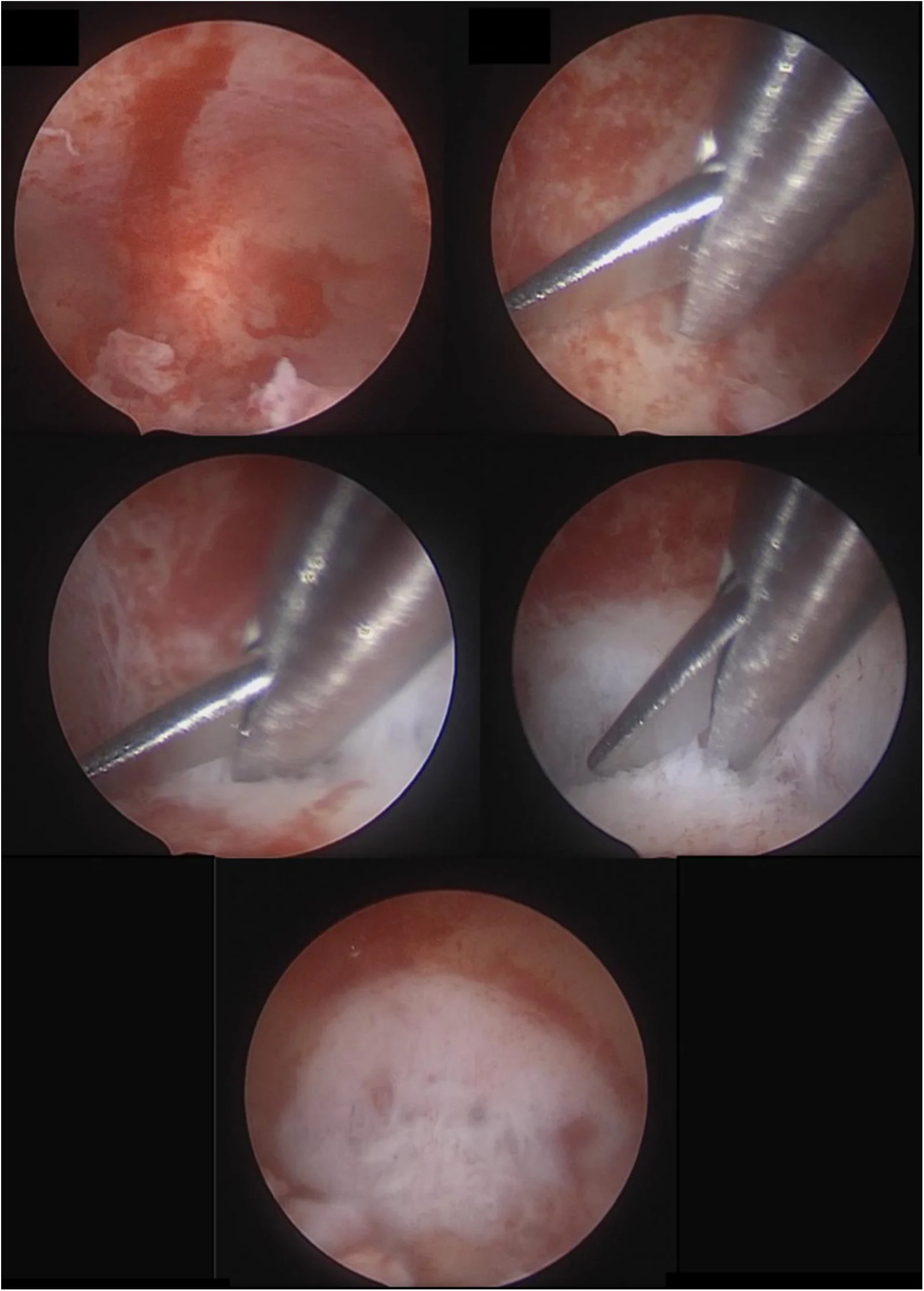
Hysteroscopic septoplasty with cold scissors. **(A,B)** Cutting the septal base. **(C,D)** Progression of septum resection. **(E)** Final view of the uterine cavity following complete septum resection.

#### 5.1.5 Tissue removal devices

Mechanical tissue removal devices have been proposed as a non-electrosurgical alternative for hysteroscopic metroplasty. Their role in congenital anomaly surgery is still limited, but they may be useful for associated endometrial thickening or focal tissue refinement. An advantage of this approach is the maintenance of a clear operative field through continuous aspiration of tissue fragments together with the absence of thermal energy, which may be desirable when preservation of the surrounding endometrium is a priority (23). **(**Figure 7**).**

**Figure 7:**
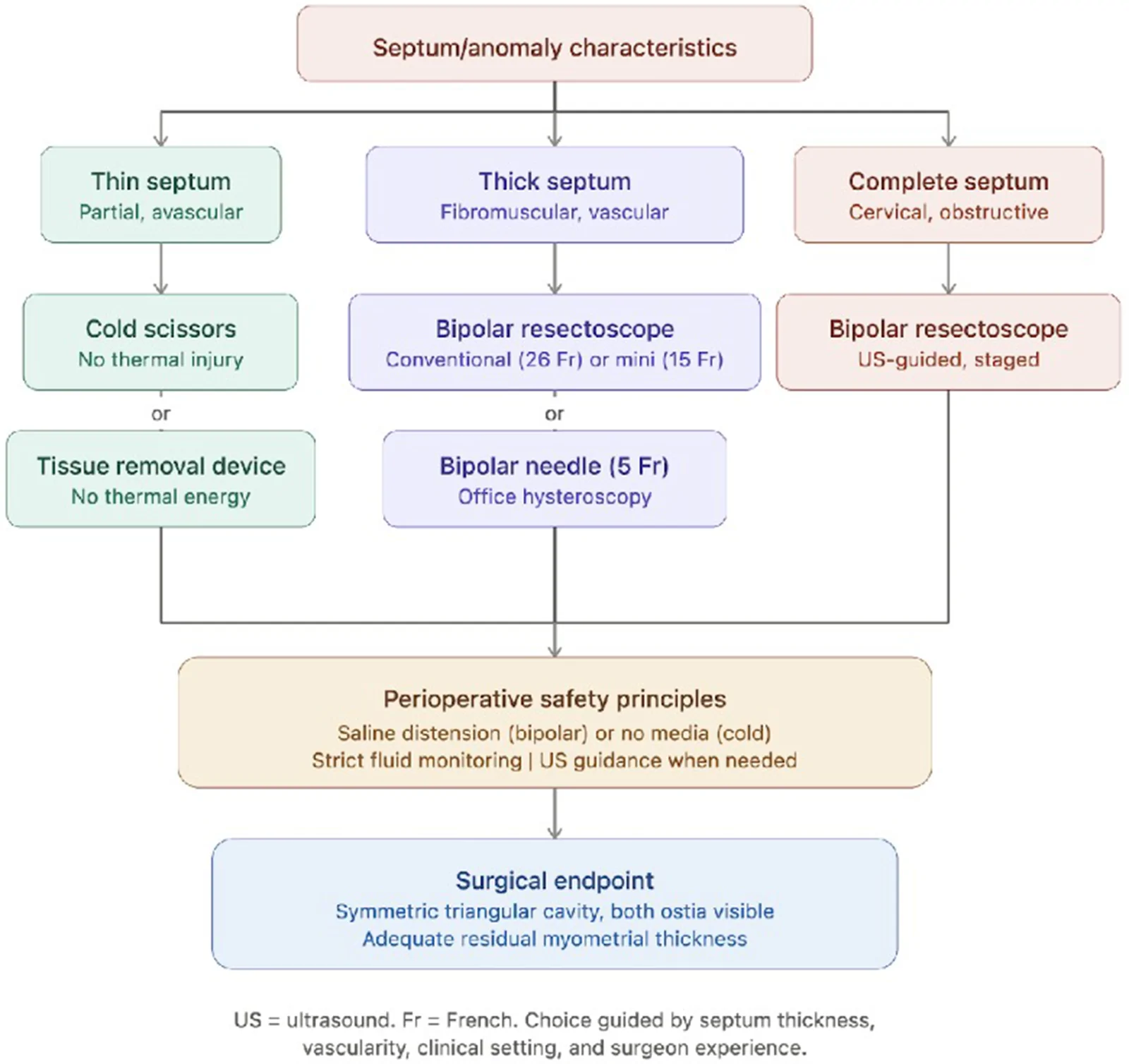
Technique and energy selection algorithm for hysteroscopic metroplasty based on septal characteristics.

### 5.2 Correction of dysmorphic uterus

A dysmorphic uterus is characterized by a normal external contour and an abnormal internal cavity, usually narrow, T-shaped, tubular, or otherwise constricted. Unlike septate uterus, the operative goal is not removal of a central septum, but controlled cavity remodeling by incising fibromuscular lateral wall constriction and, when appropriate, anterior or posterior stenotic bands (24, 25).

#### 5.2.1 Technical approach to dysmorphic uterus

Preoperative three-dimensional ultrasound, and MRI when needed, should define lateral wall thickness, cavity width, cornual anatomy, and residual myometrial safety margins. Metroplasty is typically performed by longitudinal incisions along the lateral walls near the 3 and 9 o'clock positions, extending toward the cornual regions according to the morphology. Intraoperative ultrasound is advisable in narrow cavities, severe constriction, or uncertain myometrial thickness. The endpoint is a more physiological triangular cavity with improved lateral expansion and access to both ostia, without excessive weakening of the uterine wall (9, 26) **(**Figure 8**)**.

**Figure 8:**
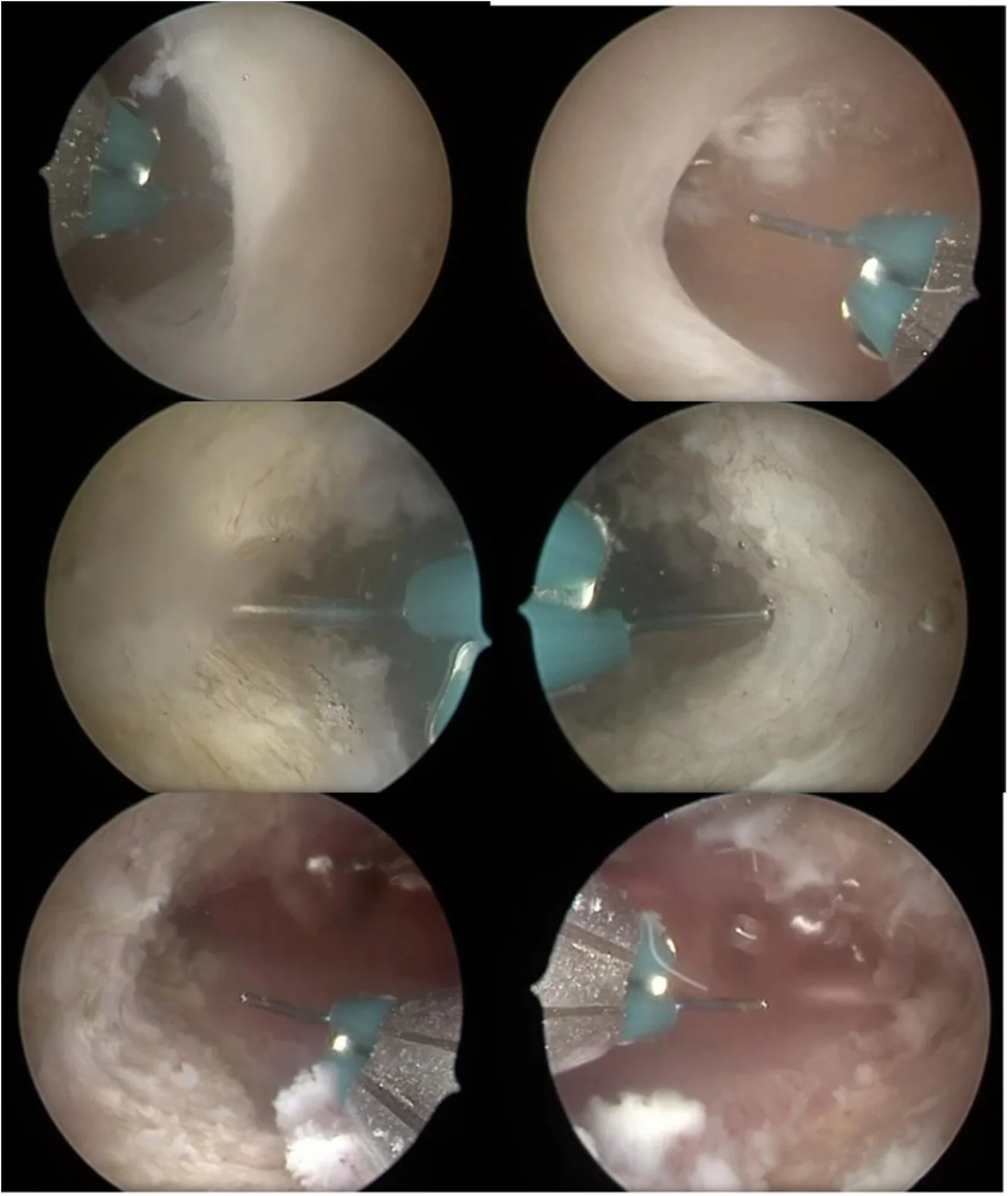
Hysteroscopic metroplasty for T-shaped uterus. **(A-D)** Lateral wall incisions using mini-resectoscope 15-Fr. **(E,F)** Progressive cavity reshaping to triangular, physiologic morphology.

The HOME-DU technique and related office-based approaches use miniaturized bipolar instruments to expand the dysmorphic cavity, often followed by anti-adhesion strategies. Observational studies and meta-analyses suggest improved reproductive outcomes after metroplasty for T-shaped uterus, with low reported complication rates, but the evidence remains heterogeneous and largely non-randomized (27–29). Therefore, routine surgery for all dysmorphic cavities cannot be recommended; patient selection should focus on women with infertility, recurrent implantation failure, or recurrent pregnancy loss after exclusion of alternative causes (13, 24, 25, 30, 31).

#### 5.2.2 U1c and Y-shaped variants

Y-shaped and other U1c variants are less well defined than T-shaped uterus. Diagnostic criteria, surgical endpoints, and reproductive indications remain insufficiently standardized. Hysteroscopic correction should be considered only in carefully selected patients with significant reproductive failure and a plausible causal relationship between cavity morphology and outcome. Incisions should be individualized, conservative, and guided by imaging to avoid unnecessary myometrial injury (28, 29).

### 5.3 Bicorporeal septate uterus

A bicorporeal septate uterus combines an external bicorporeal configuration with an internal septate component. Hysteroscopy can correct only the intracavitary septate component; it cannot alter the external uterine contour. Precise preoperative imaging is mandatory to avoid treating a bicorporeal uterus as if it were a simple septate uterus. When correction is undertaken, the objective should be clearly limited to improving the communicating cavity while preserving the myometrial safety margin (3, 10, 13).

### 5.4 Septate uterus with coexisting fundal myoma

A septate uterus with a coexisting fundal submucosal myoma is technically demanding because the septum and myoma may occupy the same fundal-cornual territory. Mapping must define the number, size, FIGO type, free myometrial margin, and spatial relationship to the septum and tubal ostia. Combined septum incision and myomectomy may increase the risk of perforation, fluid intravasation, bleeding, and postoperative adhesions; a staged strategy should be considered when operative time, fluid deficit, or visualization becomes unfavorable (32, 33).

An intraseptal myoma is rarer. A recent report described a staged hysteroscopic approach in which septal incision was performed to the myoma plane, myomectomy was completed over more than one step, and residual septal tissue was subsequently removed, with second-look hysteroscopy confirming cavity restoration and absence of adhesions (22). This example reinforces the value of staged reconstruction in complex fundal disease **(**Figure 9**).**

**Figure 9:**
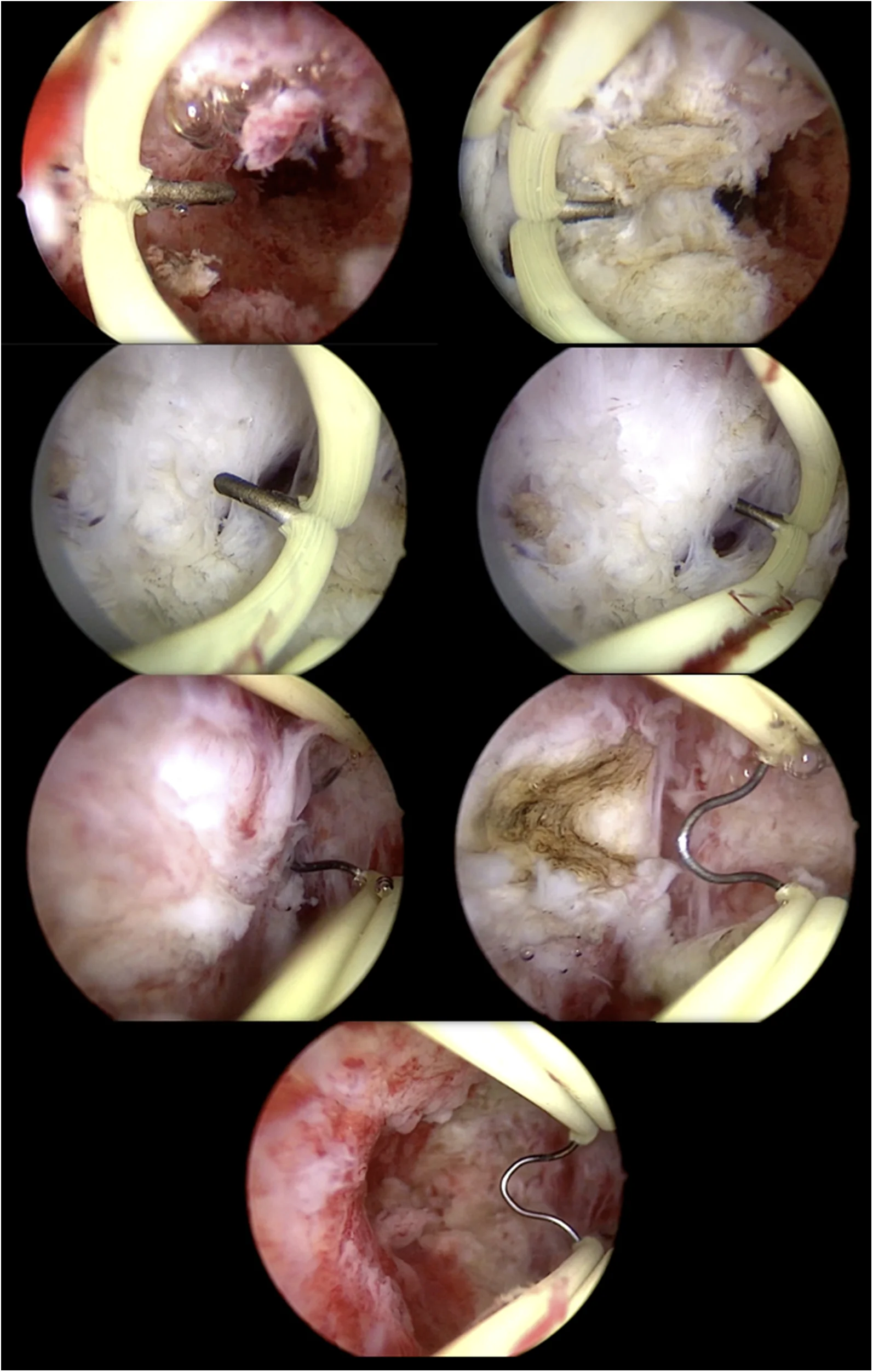
Hysteroscopic metroplasty for septate uterus with intraseptal myoma. (**A, B**) Septal incision using resectoscope and Collins electrode to the level of the myoma pseudocapsule. (**C, D**) Identification and exposure of the myoma pseudocapsule. (**E, F**) Myomectomy performed using a bipolar loop electrode. (**G**) Final hysteroscopic view demonstrating complete resection of the uterine septum and intraseptal myoma, resulting in a unified endometrial cavity.

## 6 Robert's uterus: embryology, diagnosis, and hysteroscopic management

### 6.1 Embryology and pathophysiology

Robert's uterus is a rare asymmetric septate uterus with a non-communicating blind hemicavity, an intact external fundal contour, and a contralateral hemicavity communicating with the cervix. The obstructed hemicavity contains functional endometrium, leading to cyclical pain and hematometra despite apparently normal menses through the communicating cavity. The anomaly must be distinguished from unicornuate uterus, bicornuate uterus, and complete septate uterus (2, 34, 35).

### 6.2 Diagnostic challenges and imaging

Diagnosis is frequently delayed because two-dimensional ultrasound may not identify the normal external contour and blind cavity. Three-dimensional ultrasound is the primary diagnostic tool, demonstrating the preserved external fundal contour, asymmetric septum, blind hemicavity, and hematometra. MRI provides complementary information in advanced hematometra, complex anatomy, or technically limited ultrasound. Saline infusion sonography may help delineate the communicating cavity (34–36).

### 6.3 Contemporary hysteroscopic treatment strategy

For Robert's uterus, modern treatment has shifted from laparotomy or combined laparoscopy-hysteroscopy toward ultrasound-guided hysteroscopic management in experienced centers. The surgical goal is to create communication between the blind and accessible cavities, drain hematometra, and prevent recurrence while preserving the myometrium. The diagnostic work-up and hysteroscopic management of Robert's uterus are summarized in Figure 10. Mandatory principles include preoperative three-dimensional mapping, intraoperative real-time ultrasound guidance, cautious initial incision with a fine bipolar electrode or cold technique when feasible, avoidance of unguided energy use, confirmation of drainage and cavity communication, and second-look hysteroscopy to assess healing and adhesions (34–36).

**Figure 10:**
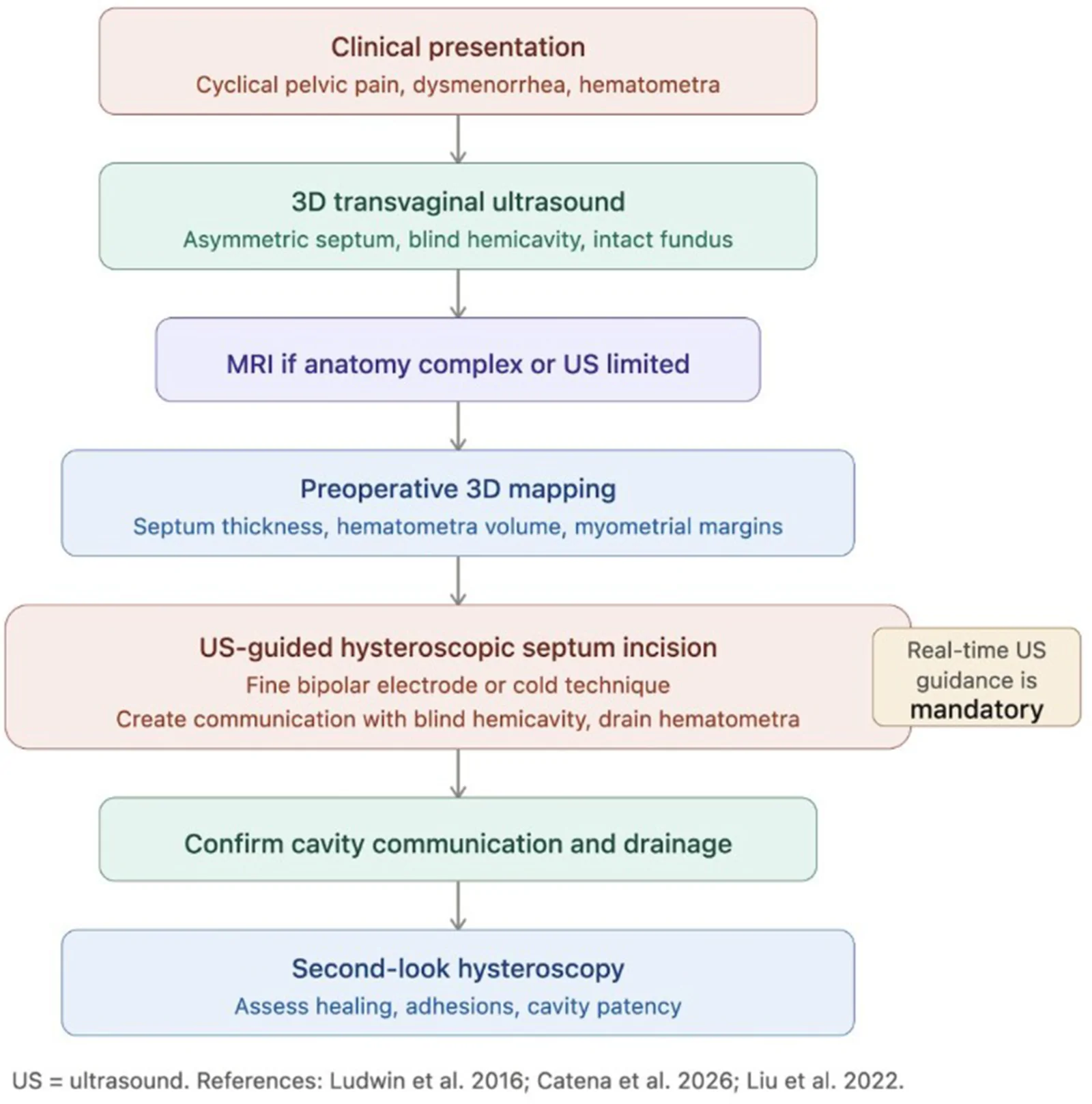
Robert's uterus: diagnosis and management.

## 7 Emerging techniques

### 7.1 Ultrasound-Guided hysteroscopy

Ultrasound-guided hysteroscopy is increasingly important in complex congenital anomaly surgery. Real-time transabdominal, transvaginal, or transrectal ultrasound can localize the instrument tip, measure residual myometrial thickness, define the fundal endpoint, and guide controlled entry into obstructed cavities. Its value is greatest in complete septa, dysmorphic uteri with narrow lateral walls, bicorporeal septate variants, Robert's uterus, and cases with prior surgery or distorted anatomy (15, 32, 34, 37).

### 7.2 Laser resection

Holmium:YAG and other laser platforms have been proposed as alternative energy sources for precise hysteroscopic incision with limited lateral thermal spread. Early reports suggest technical feasibility, including in septate uterus, but there is no high-quality evidence demonstrating superiority over bipolar electrosurgery or cold scissors for surgical, reproductive, or obstetric outcomes (38). Laser-assisted metroplasty should therefore be regarded as an option for selected settings rather than a routine standard.

### 7.3 Artificial intelligence (AI) and computer vision

Artificial intelligence-assisted hysteroscopy is a developing field with possible applications in anatomical landmark recognition, lesion detection, image-based risk prediction, myometrial safety estimation, and integration with fluid monitoring systems. Current evidence is developmental and largely outside the specific context of congenital anomaly surgery. No validated AI system has yet been established for real-time intraoperative safety control in uterine anomaly correction (5, 39, 40).

### 7.4 Robotic platforms

Robotic hysteroscopy remains experimental. Miniaturized robotic concepts may eventually offer tremor reduction, improved instrument stability, and bimanual manipulation in small intrauterine spaces, but clinical platforms, cost-effectiveness data, and outcome studies are lacking. At present, robotic hysteroscopy should be framed as a future direction only (41).

## 8 Perioperative safety

### 8.1 Energy selection

Energy selection should balance cutting efficiency, visualization, hemostasis, and endometrial preservation. Bipolar systems using saline are preferred for most operative hysteroscopic procedures, because they avoid the dilutional hyponatremia associated with monopolar systems using hypotonic media. However, saline does not eliminate volume overload; it shifts the principal risk from hypotonic hyponatremia to normonatremic hypervolemia, including pulmonary edema in severe cases (18, 42, 43).

Cold scissors are preferred when thermal injury should be minimized and when septal tissue is thin. Bipolar electrosurgery is useful for thick or vascular septa, broad fibromuscular tissue, and cases requiring reliable hemostasis. Laser technology may be used selectively, but lacks comparative superiority data (2, 40).

### 8.2 Fluid risk and deficit monitoring

Fluid risk in congenital anomaly surgery depends on the depth and surface area of exposed myometrium, cavity size, distension pressure, operative time, venous sinus exposure, and outflow accuracy. Distension pressure should be kept at the lowest level that maintains adequate visualization and ideally below mean arterial pressure. Strict fluid balance monitoring, clear thresholds for interruption, and immediate communication with anesthesia are essential. Automated deficit monitoring is preferable when available, but manual systems require disciplined measurement and awareness of common sources of error (44–46).

### 8.3 Perforation prevention and myometrial preservation in distorted anatomy

The central safety principle is to incise enough to normalize the cavity, but no further. Perforation risk increases in distorted anatomy, complete septa, obstructed cavities, dysmorphic uteri with thin lateral walls, and cases requiring energy near the fundus. Both tubal ostia should be identified before completing septal incision, and ultrasound guidance should be used liberally when the endpoint is uncertain. Staging is preferable to persisting under poor visualization, rising fluid deficit, or uncertain orientation (32, 33, 47).

Postoperative adhesions remain a concern after reconstructive hysteroscopy. Evidence for routine barriers, estrogen therapy, or second-look hysteroscopy is limited, but selected high-risk cases, such as broad septa, fundal myomectomy, repeat surgery, or extensive dysmorphic remodeling, may justify individualized adhesion-prevention strategies and planned reassessment (19, 48–52).

### 8.4 Adhesion prevention and postoperative assessment after hysteroscopic metroplasty

Postoperative intrauterine adhesion (IUA) prevention after hysteroscopic metroplasty remains controversial. The AAGL/ESGE guideline recognizes that the risk of *de novo* adhesions varies according to the type and extent of hysteroscopic surgery and states that adhesion barriers can reduce short-term IUA formation after procedures associated with endometrial injury (Level A evidence), although evidence regarding subsequent reproductive outcomes remains limited (48). Recent meta-analytic data suggest that hyaluronic acid–based gels may reduce postoperative adhesion formation, however, the available studies include heterogeneous intrauterine procedures, evidence specifically addressing septum resection remains limited, and their impact on live birth and miscarriage is still uncertain (53). The role of routine postoperative estrogen or intrauterine balloon placement after septum resection also remains unclear, with inconsistent findings across available studies. In a 2025 multicenter randomized trial including 253 women undergoing transcervical septum resection, IUA rates were comparable among surgery alone, balloon placement, and oral estrogen groups (9.0%, 7.7%, and 13.1%, respectively; *P* = 0.464), without a significant improvement in reproductive outcomes (54). Evidence supporting routine IUD placement after septum resection is likewise limited (48). Overall, no single postoperative strategy can currently be recommended for universal use after hysteroscopic metroplasty, and adhesion prophylaxis should be individualized according to the extent of endometrial injury and the estimated risk of IUA formation.

Postoperative reassessment is also relevant because uterine morphology may continue to remodel during healing, and the immediate postoperative appearance may not reflect the final anatomy. In a prospective study of 60 women undergoing hysteroscopic metroplasty, Casadio et al. demonstrated changes in fundal morphology at three-month 3D transvaginal ultrasound. Fundal morphology changed in 58% of patients, and 72% fulfilled the study criteria for consideration of a second-step metroplasty despite apparently adequate initial correction (55). These findings support delayed anatomical reassessment before further surgical intervention, although they should be interpreted cautiously given the small sample size, observational design, and lack of reproductive outcome data. Second-look hysteroscopy may provide additional value by allowing direct assessment of cavity healing and simultaneous diagnosis and treatment of early adhesions or a clinically relevant residual septum. A recent systematic review of 15 studies including 1,409 women reported that 933 underwent second-look hysteroscopy, generally within 4–20 weeks after metroplasty. Residual septa ≥1 cm were identified in 2.6%, while postoperative IUA rates varied substantially across studies (56). Given the heterogeneity of the available evidence, routine second-look hysteroscopy cannot be universally recommended and should be considered selectively. Postoperative follow-up may also incorporate 3D imaging and adhesion-prevention strategies according to individual risk.

## 9 Evidence gaps and research priorities

The evidence base remains limited by several structural problems. High-quality randomized trials are sparse, and the TRUST trial evaluated only septate uterus under specific criteria and outcome definitions. No comparable randomized evidence exists for T-shaped uterus, Y-shaped uterus, dysmorphic uterus, or Robert's uterus. Most recommendations in these areas rely on observational data, case series, and expert consensus (11, 13, 51).

Diagnostic heterogeneity is a major barrier to evidence synthesis. ESHRE/ESGE, CUME, and ASRM definitions do not identify identical populations. Future studies should report raw morphometric measurements in addition to classification labels, enabling reclassification and pooled analysis across systems (1, 10, 12).

Comparative technique data are also insufficient. There are no adequately powered trials comparing cold scissors, bipolar needle electrodes, mini-resectoscopy, conventional resectoscopy, laser, office-based treatment, and operating-room treatment using live birth and obstetric outcomes as primary endpoints. Postoperative adhesion prevention also lacks robust anomaly-specific trials (28).

Research priorities should include prospective registries and pragmatic comparative studies with standardized diagnostic criteria, clear surgical endpoints, live birth as a core outcome, long-term obstetric follow-up, and complication reporting stratified by anomaly type and technique. AI-assisted anatomical guidance and safety monitoring should be evaluated prospectively before clinical implementation (13, 40) **(**Figure 11**)**.

**Figure 11:**
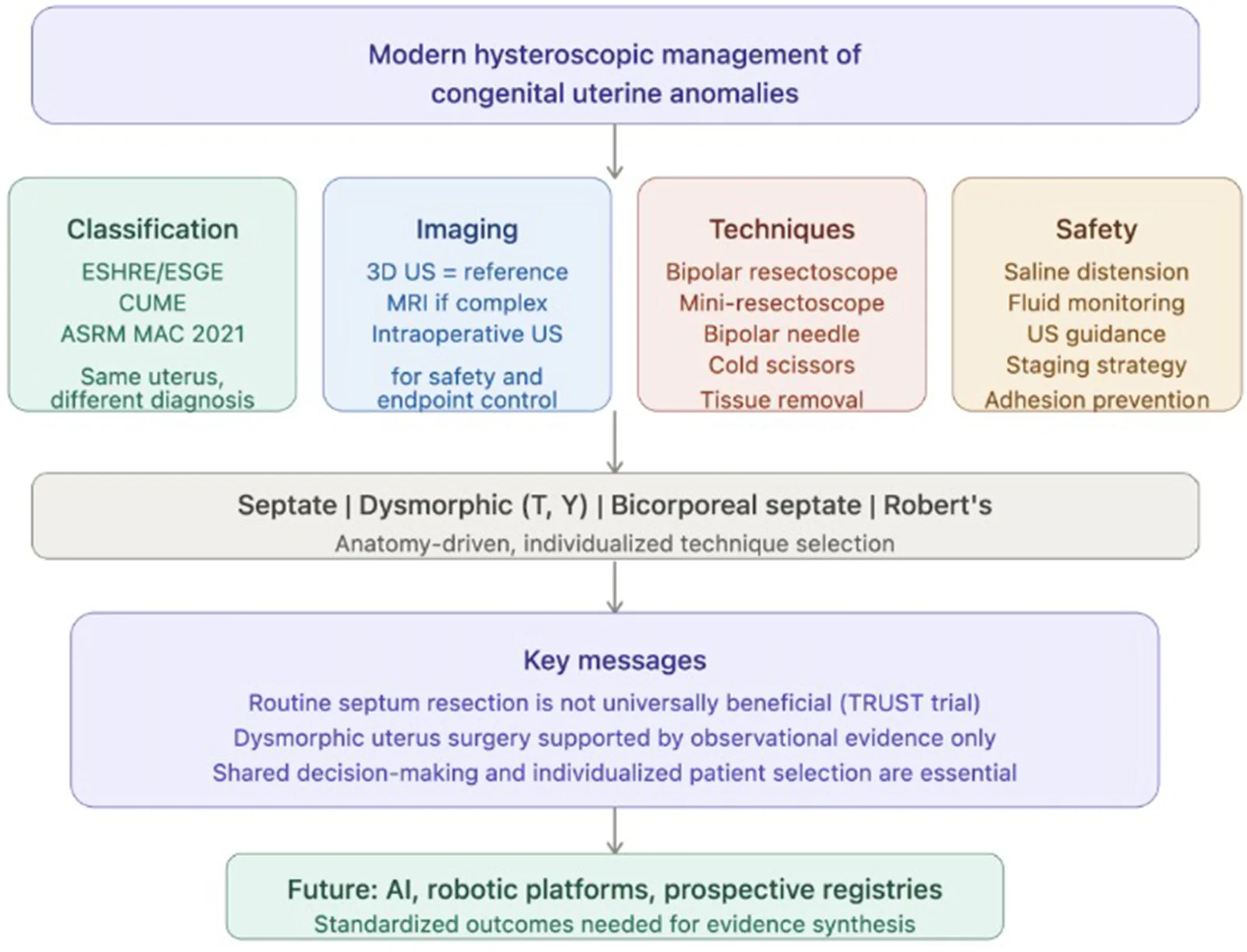
Graphical abstract summarizing key aspects of congenital uterine anomalies diagnosis and management.

## 10. Conclusions

Hysteroscopic management of congenital uterine anomalies has evolved into an anatomy-driven, conservative, and increasingly image-guided field. Bipolar saline-based platforms, miniaturized instruments, office approaches, and intraoperative ultrasound have improved precision and safety, allowing selected anomalies once treated by laparotomy to be managed hysteroscopically in experienced centers (4, 5).

These advances must be interpreted in light of persistent uncertainty. Septum resection should not be considered universally beneficial, dysmorphic uterus surgery remains supported mainly by observational evidence, and emerging technologies require prospective validation. The governing principle should be reconstructive discipline: the least necessary intervention, with maximal preservation of endometrium and myometrium, guided by high-quality imaging, individualized patient selection, and shared decision-making (11, 13).
