# Supplementary material for: Modern hysteroscopic management of congenital uterine anomalies: current techniques, emerging technologies, and future directions
Source: Front Surg. 2026 Oct 1;13:1931279. doi: 10.3389/fsurg.2026.1931279 (PMC13669504; doi:10.3389/fsurg.2026.1931279)
Supplement: Supplementary file 1 [file Supplementaryfile1.docx]

**Modern Hysteroscopic Management of Congenital Uterine Anomalies: Current Techniques, Emerging Technologies, and Future Directions**

*Vieira et al.*

Supplementary Material

Full Search Strategy:

**MEDLINE/PubMed**

("Uterine Anomalies"[Mesh] OR "Mullerian Anomalies"[Mesh] OR "uterine anomal*"[tiab] OR "Mullerian anomal*"[tiab] OR "Müllerian anomal*"[tiab] OR "Mullerian malformation*"[tiab] OR "Müllerian malformation*"[tiab] OR "septate uterus"[tiab] OR "uterine septum"[tiab] OR "dysmorphic uterus"[tiab] OR "T-shaped uterus"[tiab] OR "T shaped uterus"[tiab] OR "Y-shaped uterus"[tiab] OR "Y shaped uterus"[tiab] OR "bicornuate uterus"[tiab] OR "bicorporeal uterus"[tiab] OR "Robert's uterus"[tiab] OR "Robert uterus"[tiab]) AND ("Hysteroscopy"[Mesh] OR hysteroscop*[tiab] OR "hysteroscopic metroplasty"[tiab] OR metroplast*[tiab] OR "uterine septum resection"[tiab] OR "septum resection"[tiab] OR "septum incision"[tiab] OR "operative hysteroscopy"[tiab] OR "hysteroscopic surgery"[tiab])

**Embase**

('uterus malformation'/exp OR 'mullerian anomaly':ti,ab OR 'mullerian malformation*':ti,ab OR 'uterine anomal*':ti,ab OR 'septate uterus':ti,ab OR 'uterine septum':ti,ab OR 'dysmorphic uterus':ti,ab OR 't-shaped uterus':ti,ab OR 'y-shaped uterus':ti,ab OR 'bicornuate uterus':ti,ab OR 'bicorporeal uterus':ti,ab OR 'robert uterus':ti,ab OR 'robert''s uterus':ti,ab) AND ('hysteroscopy'/exp OR hysteroscop*:ti,ab OR 'hysteroscopic metroplasty':ti,ab OR metroplast*:ti,ab OR 'uterine septum resection':ti,ab OR 'septum resection':ti,ab OR 'septum incision':ti,ab OR 'operative hysteroscopy':ti,ab OR 'hysteroscopic surgery':ti,ab)

**Scopus**

TITLE-ABS-KEY("uterine anomal*" OR "Mullerian anomal*" OR "Müllerian anomal*" OR "Mullerian malformation*" OR "Müllerian malformation*" OR "septate uterus" OR "uterine septum" OR "dysmorphic uterus" OR "T-shaped uterus" OR "T shaped uterus" OR "Y-shaped uterus" OR "Y shaped uterus" OR "bicornuate uterus" OR "bicorporeal uterus" OR "Robert's uterus" OR "Robert uterus") AND TITLE-ABS-KEY(hysteroscop* OR "hysteroscopic metroplasty" OR metroplast* OR "uterine septum resection" OR "septum resection" OR "septum incision" OR "operative hysteroscopy" OR "hysteroscopic surgery")

**Web of Science Core Collection**

TS=("uterine anomal*" OR "Mullerian anomal*" OR "Müllerian anomal*" OR "Mullerian malformation*" OR "Müllerian malformation*" OR "septate uterus" OR "uterine septum" OR "dysmorphic uterus" OR "T-shaped uterus" OR "T shaped uterus" OR "Y-shaped uterus" OR "Y shaped uterus" OR "bicornuate uterus" OR "bicorporeal uterus" OR "Robert's uterus" OR "Robert uterus") AND TS=(hysteroscop* OR "hysteroscopic metroplasty" OR metroplast* OR "uterine septum resection" OR "septum resection" OR "septum incision" OR "operative hysteroscopy" OR "hysteroscopic surgery")

**Supplementary Figure S1: ESHRE/ESGE classification of uterine anomalies: schematic representation (Class U2: internal indentation >50% of the uterine wall thickness and external contour straight or with indentation <50%, Class U3: external indentation >50% of the uterine wall thickness, Class U3b: width of the fundal indentation at the midline >150% of the uterine wall thickness).**

Grimbizis GF, Gordts S, Di Spiezio Sardo A, Brucker S, De Angelis C, Gergolet M, et al. The ESHRE/ESGE consensus on the classification of female genital tract congenital anomalies. Hum Reprod. 2013;28(8):2032–2044. doi:10.1093/humrep/det098.
